# Prediction of FAD interacting residues in a protein from its primary sequence using evolutionary information

**DOI:** 10.1186/1471-2105-11-S1-S48

**Published:** 2010-01-18

**Authors:** Nitish K Mishra, Gajendra PS Raghava

**Affiliations:** 1 Institute of Microbial Technology, Sector 39A, Chandigarh, INDIA

## Abstract

**Background:**

Flavin binding proteins (FBP) plays a critical role in several biological functions such as electron transport system (ETS). These flavoproteins contain very tightly bound, sometimes covalently, flavin adenine dinucleotide (FAD) or flavin mono nucleotide (FMN). The interaction between flavin nucleotide and amino acids of flavoprotein is essential for their functionality. Thus identification of FAD interacting residues in a FBP is an important step for understanding their function and mechanism.

**Results:**

In this study, we describe models developed for predicting FAD interacting residues using 15, 17 and 19 window pattern. Support vector machine (SVM) based models have been developed using binary pattern of amino acid sequence of protein and achieved maximum accuracy 69.65% with Mathew's Correlation Coefficient (MCC) 0.39 and Area Under Curve (AUC) 0.773. The performance of these models have been improved significantly from 69.65% to 82.86% with MCC 0.66 and AUC 0.904, when evolutionary information is used as input in SVM. The evolutionary information was generated in form of position specific score matrix (PSSM) profile by using PSI-BLAST at e-value 0.001. All models were developed on 198 non-redundant FAD binding protein chains containing 5172 FAD interacting residues and evaluated using fivefold cross-validation technique.

**Conclusion:**

This study suggests that evolutionary information of 17 amino acid patterns perform best for FAD interacting residues prediction. We also developed a web server which predicts FAD interacting residues in a protein which is freely available for academics.

## Background

Determining function of a protein is one of the most challenging problems of the post-genomic era. In past various techniques have been developed for predicting the function of proteins using information derived from sequence similarity or clustering patterns of co-regulated genes, interaction of protein etc. It is important to understand interaction of protein with other proteins or ligands in order to understand it function. One of most important ligands among the molecules that interact with proteins is nucleotide. Prediction of proteins and nucleotide interaction can be divided in two categories (I) short nucleotide interaction, where short nucleotide (mono/di/trinucleotide) interacts with proteins (II) polynucleotide interactions, where polynucleotide (DNA/RNA) interacts with proteins.

Many proteins use small nucleotides as a source of energy and signaling molecules inside the cell (adenine and guanine nucleotides). The flavin and nicotinamide nucleotides work as electron donor/acceptors in lots of cellular metabolic reactions. FAD is a redox cofactor involved in several important reactions in metabolism. Living organism mostly generate energy by using glucose or fat molecules, both metabolic pathway regulated by enzyme which prosthetic group is FAD. Thus, identification of FAD interacting residue (FIR) is very important in molecular recognition. Despite tremendous progress in the annotation of protein, there is no any existing online tools are available for the prediction of FIR using primary sequence. Experimental method to identify FIR is very difficult and time consuming process and also very costly. We can easily identify either FAD interact with protein or not by using absorption spectra but can't identify which residues are FIRs.

In the past large number of tools have been developed for the prediction of polynucleotide (DNA/RNA) interacting residues using different machine learning techniques [1-7]. In contrast there has been only one preexisting method available for the prediction of small nucleotide-protein interaction, developed by Saito et al [8]. They developed method for small nucleotide binding site prediction using empirical score approach and detect 40% FAD binding sites correctly. Saito et al. methods only give us idea about binding site but can't give information about the FAD interacting residues. Kallberg et al. [9] used simple sequence in Hidden Markov Model and developed method for identifying Rossmann folds and predict there coenzyme specificity (NAD, NADP, FAD) and found that FAD least preferred cofactor. So there studies suggest that FAD interacting residues can't predict easily. Thus, the development of computational method for predicting FIR in a protein from its amino acid sequence is very important for functional annotation of proteins.

In this work, a systematic attempt has been made to predict FIRs in a protein sequences using binary pattern and PSSM profiles of 5172 FIRs and non-FIRs of 198 non-redundant protein chains. In first step FBP chain were analyzed, then SVM model were developed by using binary pattern of FIRs. It have been observed in past that evolutionary information provide more information, thus we developed SVM models using PSSM profiles obtained from PSI-BLAST [10]. All models developed in this study were evaluated using five-fold cross validation technique. FADPred can directly predict the FAD interacting residues using binary pattern of sequence and its evolutionary information. Our server will be useful for experimental biologists working on flavoproteins/flavoenzymes.

## Methods

### Dataset

In first step of data collection we use SuperSite documentation [11] and extract 675 PDB IDs of protein having contact with FAD in PDB. We download the sequence of all chains of these PDB IDs using web download section in PDB. In next step we use these PDB IDs in Ligand Protein Contact (LPC) [12] and get total 1539 chain which interacts with FAD with their corresponding interacting residues and its position. Then we remove redundant chains which have more than 40% similarity by using CD-HIT [13], finally retrieved a total 198 interacting chains with a total 5172 FIRs remaining all residues are non-FIRs. In this study we used 5172 FIRs and 5172 non-FIRs for developing our models. Sequences of these 198 FBP with their PDB ID and chain name are freely available [14], where FIRs are in lowercase and non-FIRs are in uppercase.

### Five-fold cross-validation

Fivefold cross-validation technique has been used to evaluate the performance of all the models developed in this study. In this technique dataset is randomly divided into five sets where each set consist of nearly equal number of interacting and non-interacting patterns out of these five sets four sets are used for training and the remaining set for testing. This process is repeated five times in such a way that each set is used once for testing. The final performance is obtained by averaging the performance of all the five sets.

### Pattern or window size

We generated an overlapping pattern of 17 residues, for each FAD interacting chains sequences. If the central residue of pattern was FIR, then we classified the pattern as positive or FIR pattern, otherwise it was termed as non-interacting or negative pattern. In this study we follow the similar approach adopted by Kaur and Raghava [15-17] for prediction of turns in protein sequences. In additional to 17 residue window we also generate pattern of 15 and 19 residues. In this study we used unique residue patterns for binary and PSSM pattern generation. Finally we have total 4896, 4974 and 4974 unique pattern for interacting residues respectively in 15, 17 and 19 residue window, and randomly picked equal number of non-interacting pattern as negative data.

### Support Vector Machine (SVM)

An excellent machine learning technique SVM [18] has been used for the prediction of FIRs. All SVM models have been developed by using a freely available package SVM_light [19]. The SVM is particularly attractive to biological sequence analysis due to its ability to handle noise, dataset and large input space. Further details about SVM can be obtained from Vapnik's [20] paper. The software allows users to run SVM using various numbers of parameters as well as to select inbuilt kernel functions, including a linear, polynomial and Radial Basis Function (RBF).

### Evolutionary information

This was obtained from position-specific scoring matrix (PSSM) generated from PSI-BLAST search against non-redundant (nr) database [21] of protein sequences. The PSSM matrix was generated by three iterations of searching at cutoff *e*-value of 0.001 for inclusion of sequences in next iteration. The generated PSSM contained the probability of occurrence of each type of amino acid at each position along with insertion/deletion. Hence, PSSM is considered as a measure of residue conservation in a given location. This means that evolutionary information for each amino acid is encapsulated in a vector of 20 dimensions where the size of PSSM matrix of a protein with *N *residues is 20 × *N*. Where 20 dimension are 20 standard amino acids. We normalized each value within 0-1 range using equation:

Where val is the PSSM score and Val is its normalized value.

### Figure of merits

In this study performance of constructed modules has been evaluated by using five-fold cross-validation techniques. Following threshold dependent parameters: sensitivity (*Sn*) or percent coverage of FIR is the percentage of FIR residue predicted as FIR; specificity (*Sp*) or percent coverage of non-interacting residues is the percentage of non-FIR predicted as non-FIR; overall accuracy (*Ac*) is the percentage of correctly predicted interacting residues has been used for assessing the performance of method. These parameters can be using following equations:(1)(2)(3)(4)

Where TP is correctly predicted FIRs, TN is correctly predicted non-FIRs, FP is the number of non-FIRs predicted as FIR and FN is the number of FIRs wrongly predicted as non-FIR. Matthew's correlation coefficient (*MCC*) equal to 1 is regarded as a perfect prediction, whereas 0 is for completely random prediction. We also calculated AUC of ROC plot which is a threshold independent parameter.

### Description of web server

The prediction method described in this paper is implemented in the form of a web-server FADPred [22]. The common gateway interface (CGI) script of FADPred is written using PERL version 5.03. FADPred server is installed on a Sun Server (420E) under UNIX (Solaris 7) environment. It is a user-friendly web server which allows users to submit their protein sequence in two different ways; first browse and upload the fasta sequence file and second, either type or paste fasta sequence in a box which is available on submit page. This server allows users to predict FAD binding residues using both binary pattern and PSSM based SVM models with different threshold range from -1 - +1. Here we provide option for both binary pattern and PSSM user can select according to their choice and get the result through mail also. The default method is PSSM and threshold is 0.0, sensitivity and specificity is roughly found to equal during the five-fold cross-validation procedure at this threshold. The prediction result presented in graphical form where the predicted FIRs and non-FIRs are displayed in different color. We are using PSSM as default option and it takes several minutes to predict FAD interacting residues in a protein.

## Results

### Compositional analysis

**Figure 1:**
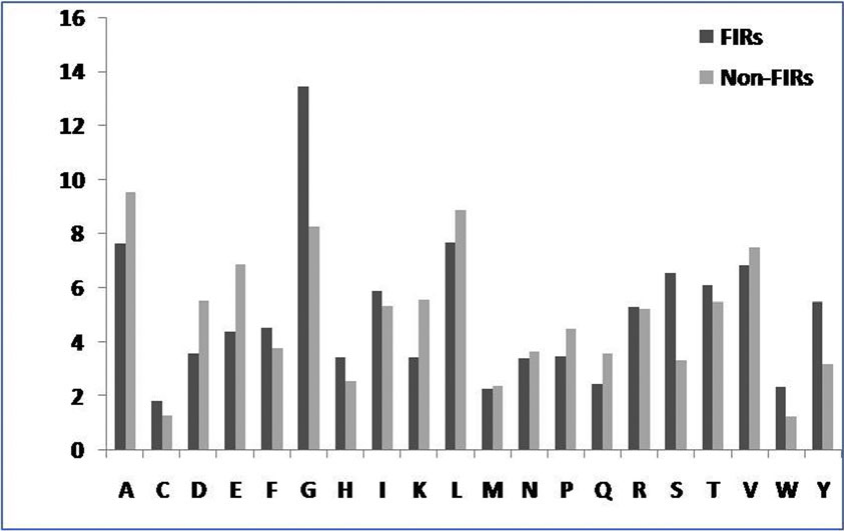
**Percentage composition of interacting and non-interacting residues**.

We calculated the percentage composition of interacting and non-interacting residues and found Gly, Tyr and Ser residues were more abundantly interact with FAD as compare to non-interacting residues (Figure 1). The dominance of these residues shows a vital role of these residues in FAD interaction.

### SVM model using binary pattern of amino acid sequence

In our study we generated multiple 17 residue long window for negative and positive pattern. These sequence patterns were converted to binary patterns, where a pattern of length *N *was represented by a vector of dimension *N *× 21 and each amino acid in that pattern was represented by a vector of 21 (e.g. Ala by 1,0,0,0,0,0,0,0,0,0,0,0,0,0,0,0,0,0,0,0) which contained 20 amino acids and one dummy amino acid X. As shown in Table 1, this SVM-based model was able to give a maximum MCC of 0.39 with 69.65% accuracy having minimum difference in sensitivity and specificity. Threshold at which sensitivity and specificity is nearly same is shown by bold font, in order to balance sensitivity and specificity. Similarly we achieved accuracy 70.31% with MCC of 0.41 for 15 window patterns and accuracy 70.49 with MCC of 0.41 for 19 window patterns. AUCs are 0.769, 0.773 & 0.770 respectively for 15 window, 17 window and 19 window pattern models (Table 2). The performances of models were evaluated at residue level.

**Table 1:** The performance of SVM model using binary pattern. Bold values indicate the point where sensitivity and specificity is equal or minimum difference with highest MCC.

15 window					17 window				19 window			
**Thes**	**Sen**	**Spe**	**Acc**	**MCC**	**Sen**	**Spe**	**Acc**	**MCC**	**Sen**	**Spe**	**Acc**	**MCC**

-1.0	100	0	50.0	0.0	87.49	42.38	64.94	0.33	99.98	0.06	50.02	0.01
-0.9	100	0	50.0	0.0	86.57	44.27	65.42	0.34	99.96	0.32	50.14	0.03
-0.8	100	0	50.0	0.0	85.83	46.08	65.95	0.35	99.92	1.11	50.51	0.07
-0.7	100	0.04	50.02	0.01	84.96	47.89	66.43	0.35	99.68	2.45	51.07	0.09
-0.6	99.98	0.20	50.09	0.03	84.00	49.66	66.83	0.36	99.08	5.37	52.22	0.13
-0.5	99.75	1.21	50.48	0.06	82.95	51.63	67.29	0.36	97.75	11.68	54.71	0.19
-0.4	99.08	4.43	51.76	0.11	81.85	53.48	67.66	0.37	95.74	20.06	57.90	0.24
-0.3	96.98	14.26	55.62	0.20	80.76	55.19	67.97	0.37	91.64	32.39	62.01	0.30
-0.2	90.34	33.70	62.02	0.29	79.73	57.00	68.37	0.38	85.52	47.37	66.45	0.36
-0.1	78.21	58.19	68.20	0.37	78.49	58.73	68.61	0.38	76.76	61.36	69.06	0.39
**0**	**62.36**	**78.27**	**70.31**	**0.41**	77.00	60.57	68.79	0.38	**66.26**	**74.71**	**70.49**	**0.41**
0.1	46.81	90.16	68.48	0.41	75.67	62.40	69.04	0.38	55.47	83.90	69.68	0.41
0.2	33.11	95.75	64.43	0.37	74.57	63.89	69.23	0.39	45.05	90.77	67.91	0.40
0.3	23.90	98.04	60.97	0.33	73.52	65.42	69.47	0.39	34.78	94.93	64.86	0.37
0.4	17.14	99.20	58.17	0.29	72.11	67.09	69.60	0.39	26.46	97.15	61.80	0.33
**0.5**	11.83	99.65	55.74	0.24	**70.53**	**68.73**	**69.65**	**0.39**	19.78	98.39	59.09	0.29
0.6	8.58	99.84	54.21	0.21	68.80	70.23	69.51	0.39	14.21	99.34	56.78	0.26
0.7	5.84	99.92	52.88	0.17	67.11	71.83	69.47	0.39	9.93	99.74	54.84	0.22
0.8	3.96	99.94	51.95	0.14	65.34	73.34	69.34	0.39	6.41	99.86	53.14	0.18
0.9	2.12	99.98	51.05	0.10	63.77	74.45	69.26	0.39	4.06	99.92	51.99	0.14
1.0	1.27	100	50.63	0.08	62.08	76.20	69.14	0.39	2.43	99.94	51.19	0.11

**Table 2:** SVM parameters and AUC for our best models. The SVM parameter d (in polynomial kernel), g (in RBF kernel), c: parameter for trade-off between training error & margin, j: cost-factor.

Window		SVM parameter	AUC
**15 window**	Binary	d: 4 c: 1 j: 1	0.769
	PSSM	d: 5 c: 1 j: 1	0.878
**17 window**	Binary	g: 0.1 c: 2 j: 1	0.773
	PSSM	d: 4 c:5 j: 1	0.904
**19 window**	Binary	d: 3 j: 1 c: 1	0.770
	PSSM	d: 5 c: 1 j: 1	0.876

### SVM model using evolutionary information

In the past, it has been shown in several studies that evolutionary information obtained using multiple sequence alignment provides more comprehensive information about the protein than a single sequence [1,6,15-17]. In this study, the evolutionary information obtained from PSSM generated from PSI-BLAST profiles was used for predicting FIRs. As shown in Table 3, performance increased significantly when PSSM was used as input instead of single sequence. A maximum MCC of 0.62 was achieved with 80.82% accuracy using evolutionary information. Similarly we achieved accuracy 80.29 with MCC of 0.61 for 15 window patterns and accuracy 80.39 with MCC of 0.61 for 19 window patterns. AUCs are 0.878, 0.904 & 0.876 respectively for 15 window, 17 window and 19 window pattern models (Table 2).

**Table 3:** The performance of SVM model using evolutionary information. Bold values indicate the point where sensitivity and specificity is equal or minimum difference with highest MCC.

15 window					17 window				19 window			
**Thes**	**Sen**	**Spe**	**Acc**	**MCC**	**Sen**	**Spe**	**Acc**	**MCC**	**Sen**	**Spe**	**Acc**	**MCC**

-1.0	98.76	12.48	55.62	0.22	98.41	21.14	59.78	0.31	98.67	13.31	55.99	0.23
-0.9	98.38	15.41	57.09	0.25	97.98	25.91	61.95	0.34	98.12	16.81	57.46	0.26
-0.8	97.71	20.59	59.15	0.29	97.37	30.92	64.15	0.38	97.60	21.38	59.49	0.29
-0.7	96.86	25.68	61.27	0.32	96.25	37.26	66.75	0.42	96.78	26.03	61.41	0.32
-0.6	95.82	31.46	63.64	0.36	95.27	43.43	69.35	0.45	95.78	32.19	63.99	0.36
-0.5	94.51	38.63	66.57	0.40	94.26	49.72	71.99	0.49	94.51	38.81	66.66	0.40
-0.4	93.03	45.54	69.28	0.44	92.87	56.94	74.90	0.53	93.00	46.04	69.52	0.44
-0.3	91.20	53.32	72.76	0.48	91.36	63.94	77.65	0.58	91.34	54.31	72.82	0.49
-0.2	88.83	62.53	75.68	0.53	89.35	70.79	80.07	0.61	88.98	62.32	75.65	0.53
-0.1	85.47	70.86	78.16	0.57	86.70	77.08	81.89	0.64	85.64	70.92	78.28	0.57
**0**	**80.85**	**79.73**	**80.29**	**0.61**	**83.36**	**82.36**	**82.86**	**0.66**	**81.86**	**78.93**	**80.39**	**0.61**
0.1	75.62	87.72	81.67	0.64	79.20	87.39	83.30	0.67	76.12	86.05	81.09	0.62
0.2	68.59	92.60	80.59	0.63	74.84	91.83	83.34	0.68	69.28	91.42	80.35	0.62
0.3	63.42	94.75	79.08	0.61	69.61	94.60	82.10	0.66	63.44	93.96	78.70	0.60
0.4	57.60	96.17	76.88	0.58	64.86	96.05	80.45	0.64	58.06	95.66	76.86	0.58
0.5	51.38	97.22	74.30	0.55	60.07	97.29	78.68	0.62	51.18	96.85	74.01	0.54
0.6	44.20	97.93	71.07	050	54.55	98.09	76.32	0.58	44.01	97.85	70.93	0.50
0.7	36.42	98.52	67.47	0.45	47.38	98.66	73.02	0.54	35.65	98.44	67.05	0.44
0.8	28.11	98.87	63.49	0.38	38.40	99.06	68.73	0.47	27.50	98.77	63.14	0.37
0.9	20.29	99.03	59.66	0.31	28.70	99.35	64.03	0.40	19.19	99.08	59.13	0.30
1.0	12.99	99.37	56.18	0.25	19.14	99.57	59.76	0.32	11.33	99.55	55.44	0.23

## Discussion

Due to the vital roles of FAD binding proteins in cellular metabolism and difficulties in *iv-vitro *analysis or identification of FIRs, by biophysical method, there is as urgent need for computational method to identify FAD binding sites on the basis of amino acid sequence of a protein. In this direction, we had followed a systematic attempt to develop a highly accurate and robust method for predicting FAD binding residues in protein sequences. There is no any preexisting online method in our knowledge for the prediction of FIRs using primary sequence. So first of all we developed method for predicting FIRs using sequence of FBP proteins. For this study firstly we collect the information of FAD binding proteins PDB IDs with SuperSite, fasta sequence from PDB and FAD interacting residues using LPC. Then analyze FIRs and found that there is significant difference in interacting residues as well as flanking residues.

It has been reported in some of the earlier studies that SVM perform better than any other artificial intelligence (AI) techniques in interacting residue prediction. First we developed SVM model based on binary patterns of amino acid sequence. Manish et al. 2008 showed that evolutionary information perform better than simple sequence information in interacting residue prediction. Further we used evolutionary information to generate PSSM profile as input for SVM model and check overall performance of FIRs prediction. SVM parameter for each model with their AUC is given in Table 2. One of the obvious questions is why we can't use BLAST for predicting FIRs. Thus we also make an attempt to predict FAD interacting residues using BLAST and achieved very poor performance (data not shown). We also provide a direct access of our developed prediction method to public, through web server FADPred. FADPred allow users to predict FAD interacting residues in their protein sequence.

## Conclusion

**Figure 2:**
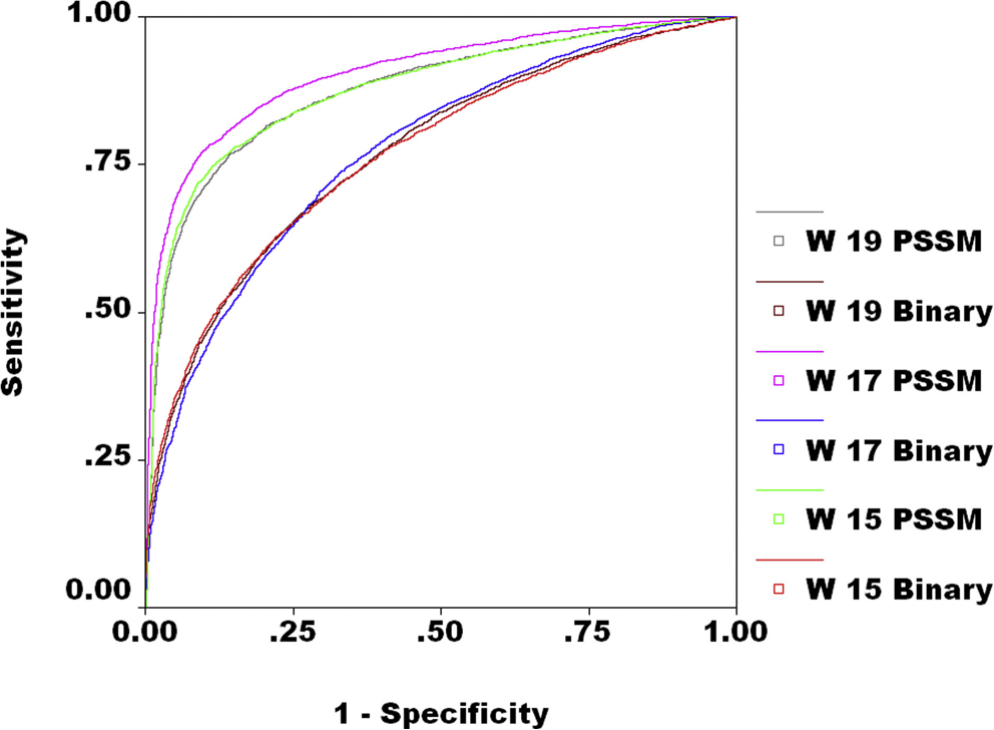
**ROC plot for 15, 17 and 19 windows size binary and PSSM models**.

In this study first, time a highly robust method has been developed to predict FAD interacting residues from protein sequence using AI technique, SVM. This study demonstrates that PSSM based method performs better than simple sequence based method. In this study we also observed that 17 window pattern perform better than 15 and 19 window pattern (Table 2, Figure 2). This study will be helpful for biologist in proteome annotation. One of the major advantage of this study is that we developed free web server; FADPred. Our web server allows users to identify FAD interacting residue in given sequence using the model trained on our data set.

## Competing interests

The authors declare that they have no competing interests.

## Authors' contributions

Mishra NK carried out the data analysis and interpretation, developed computer programs, wrote the manuscript and developed the web server and created clean datasets. GPSR conceived and coordinated the project, guided its conception and design, helped in interpretation of data, refined the drafted manuscript and gave overall supervision to the project. All authors read and approved the final manuscript.
